# Bilateral primary histiocytoid eccrine sweat gland carcinoma of eyelids^☆^

**DOI:** 10.1016/j.bjorl.2016.01.006

**Published:** 2016-04-16

**Authors:** Malgorzata Seredyka-Burduk, Pawel Krzysztof Burduk, Magdalena Bodnar, Grazyna Malukiewicz, Andrzej Kopczynski

**Affiliations:** a Nicolaus Copernicus University in Toruń, Faculty of Medicine, Department of Optometry Collegium Medicum, Toruń, Poland; b Nicolaus Copernicus University in Toruń, Faculty of Medicine, Department of Ophthalmology Collegium Medicum, Toruń, Poland; c Nicolaus Copernicus University in Toruń, Faculty of Medicine, Department of Otolaryngology and Laryngological Oncology Collegium Medicum, Toruń, Poland; d Nicolaus Copernicus University in Toruń, Faculty of Medicine, Department of Clinical Pathomorphology Collegium Medicum, Toruń, Poland

## Introduction

Primary eccrine carcinomas of the eyelid are rare. There are three types of carcinomas: mucinous, adenoid cystic, and ductal.^1^, ^2^, ^3^, ^4^, ^5^ Ductal carcinomas are classified into two subtypes: signet ring carcinoma (well-differentiated) and histiocytoid type (poorly-differentiated).^1^, ^2^, ^3^, ^5^ Primary histiocytoid eccrine sweat gland carcinoma of the eyelid is very rare and so far 13 cases have been reported.^1^, ^2^, ^3^, ^5^, ^6^, ^7^

This kind of carcinoma involves the dermis and subcutis of the eyelid and has often been misinterpreted as an inflammatory process.^1^, ^5^ It occurs in the eyelid of the elderly in one side.^1^, ^2^, ^7^ This is the first report of confirmed bilateral histiocytoid eccrine sweat gland carcinoma.

## Case report

**Figure 1:**
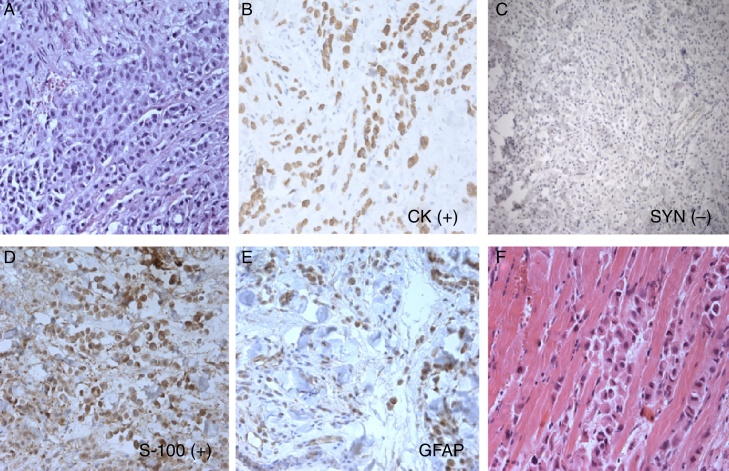
Histopathology and immunohistochemical stain, histiocytoid eccrine carcinoma: (A), cords and small nests of histiocytoid cells; tumor cells are positive for cytokeratins (B) and negative for synaptophysin (C), tumor cells are S-100 positive (D), some cells are GFAP positive (E), and cords of histiocytoid cells are separating muscle fibers (F).

**Figure 2:**
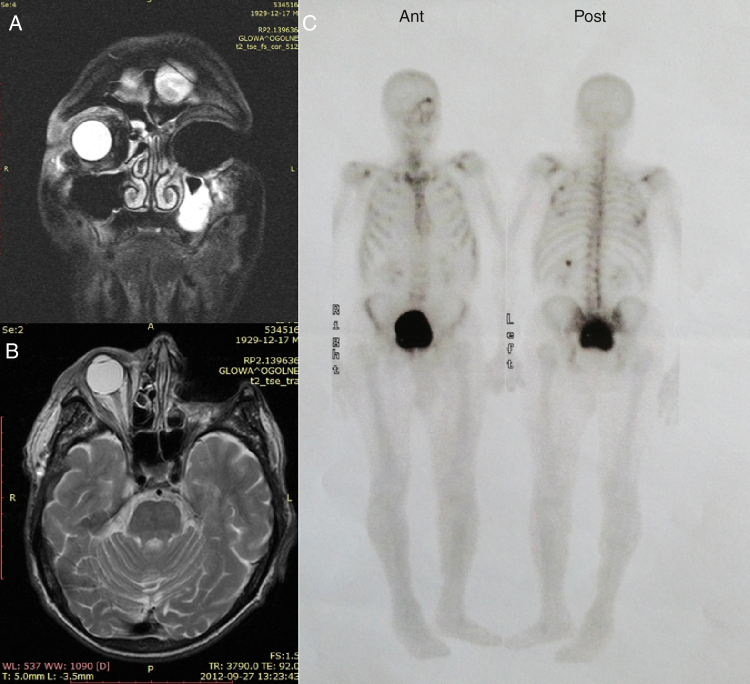
Magnetic resonance imaging scan: left orbit after exenteration without pathologic enhancement, mucus fluid in left maxillary sinus-frontal and plain projection (A and B). Bone scintigraphy, multiple intense enhancement in left orbit, and bony skeleton due to metabolic and postoperative changes (C).

**Figure 3:**
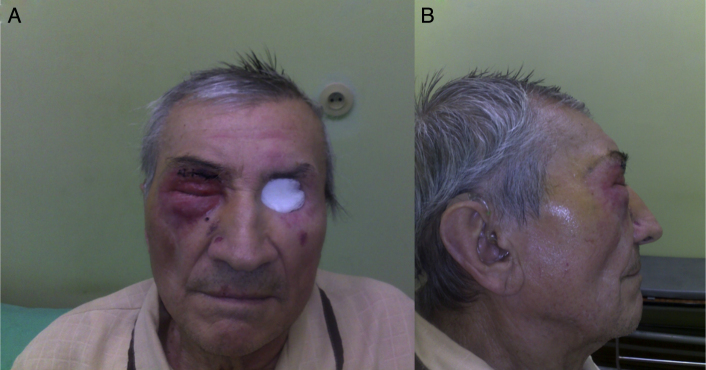
Patient two years after left orbit exenteration and radiotherapy; the second tumor is infiltrating the right periorbital skin and eyelids, frontal and lateral view (A and B).

An 80 years old man was referred to the ophthalmology department with an inflammation of the left lower eyelid that had developed at the beginning of 2009. A computed tomography (CT) without contrast enhancement showed increased thickness of the upper and lower eyelids up to 7–10 mm with abnormal soft tissue mass in the upper-medial part of the left orbit. For two months the patient had experienced a slowly growing tumor and infiltration of the left lower eyelid. The skin of the involved area seemed erythematous and asymptomatic. A biopsy of the thickened area showed a benign process with fibroblasts cells infiltration. Chest X-ray and abdominal ultrasound were normal. A standard local and oral anti-inflammatory treatment with prednizone 20 mg daily and dexamethasone eye drops two times daily was prescribed. During the two months, the patient had experienced a slowly growing tumor and infiltration of the left lower eyelid. The skin of the involved area appeared erythematous and asymptomatic. An open biopsy was then performed, showing a benign process-neuronaevus. In the following two months, the left lower eyelid showed increased diffuse thickening. A control CT presented thickness of the left lower eyelid of 7 mm and abnormal, and 15–10 mm of soft tissue in the left orbit at the same location as the previous CT. A complete excision of the left lower eyelid with reconstruction was done. Histopathological examination under microscope determined that there were cords of neoplastic cells, which were found between muscle fibers. The tumor cells had small vacuoles within the cytoplasm. There was mild pleomorphism of cell nuclei; in some nuclei, vesicles were observed. The tumor cells were positive for cytokeratins and S-100 protein and negative for synaptophysin. GFAP immunostaging gave artificial results. Morphology and clinical data were consistent with diagnosis of histiocytoid eccrine carcinoma (Fig. 1). The patient was admitted for oncologic treatment. 45 Gy of intensity-modulated radiation therapy (IMRT) were administered. Several weeks after radiotherapy, it was observed an increased diffuse thickening of the upper and lower eyelids and the ptosis. Magnetic resonance imaging (MRI) and CT showed infiltration of both eyelids and orbit with extraocular muscles involvement. A second large biopsy was done. The histopathology examination revealed histiocytoid eccrine carcinoma (Fig. 1). Orbital exenteration and wide excision was performed at the Department of Otolaryngology and Laryngological Oncology. A follow-up with positron emission tomography (PET/CT) scan, MRI, and bone scintigraphy showed no recurrences (Fig. 2). After 24 months of initial treatment, the patient started to suffer from inflammation and edema on the right eyelid (Fig. 3). CT and MRI of the right orbit showed increased thickness of the upper and lower eyelids with orbital involvement. A biopsy of the eyelids showed the same type of pathology previously diagnosed in the left eye (histiocytoid eccrine carcinoma; Fig. 1). The patient was admitted for oncologic treatment. An E9MeV, 45 Gy radiotherapy was administered. Two years later, the patient is alive without evidence of recurrence or metastatic disease.

## Discussion

The histology of ductal adenocarcinoma of the breast, eccrine sweat glands, and lacrimal or salivary gland adenocarcinoma may be identical.^1^, ^2^, ^3^, ^4^, ^8^ The histogenesis of primary cutaneous histiocytoid sweat gland carcinoma remains controversial.^4^, ^5^ The exclusive location in the axilla and eyelid with cutaneous areas containing apocrine glands supports its apocrine differentiation.^5^, ^9^

The diagnosis of primary histiocytoid variant of eccrine sweat gland carcinoma is sometimes difficult; immunohistochemical staining for CEA, cytokeratins, epithelial membrane antigen, and others, are still used.^3^, ^4^ Usually, this type of carcinoma involves the dermis and subcutis of the eyelid but, occasionally, it can be observed in the orbit or infiltrate the orbit.^3^, ^10^ The neoplastic cells involve the dermis of the eyelid and are often mistaken histopathologically with inflammatory processes or with benign neoplasms, such as granular cell tumor, melanocytic nevus, or neuronevus.^1^, ^5^, ^6^ In some cases, it could delay the diagnosis and lead to slow or fast growth of the tumor and spread of the disease, as observed in the present case.

The typical clinical findings are progressive painless infiltration of both eyelids or growth starting in one eyelid, later involving the other. The infiltration of the eyelids results in an monocle-like appearance.^2^, ^3^, ^5^, ^7^, ^9^ Diagnosis arises from careful CT and MRI of the orbit and proper histologic investigation. In case of suspicion of misdiagnose, secondary biopsy and scans should be performed immediately. Histologic differential diagnosis includes cutaneous neoplasms that may contain signet ring cells, such as basal and squamous cell carcinoma, melanocytic tumors, cylindroma or lymphoma, which are consistent with eyelid development.^5^, ^8^

Treatment of primary histiocytoid eccrine sweat gland carcinoma of the eyelid requires excision with wide margins and sometimes orbital exenteration.^5^ Adjuvant therapy includes radiotherapy and chemotherapy with 5-fluorouracil.^5^, ^7^ In some cases, a rapid progression of the tumor after surgical resection can be observed.^1^ The same situation can occur after radiotherapy alone. In the present case, the tumor was unresponsive to radiotherapy and continued to grow. A wide excision with orbital exenteration and postsurgical radiotherapy was performed, which was useful for local control. Unfortunately, a second primary histiocytoid eccrine sweat gland carcinoma of the right eyelid was diagnosed. At that time, the tumor was successfully treated with radiotherapy. It is unknown whether the second malignancy, after two years, was radiotherapy-dependent or unrelated.

The clinical course of primary histiocytoid eccrine sweat gland carcinoma is usually protracted for several years.^9^ Recurrences can be expected and metastases can reach lymph nodes, parotid gland, skin, lung, or liver.^4^, ^5^, ^7^, ^9^ To the best of the authors’ knowledge, this is the first case of bilateral histiocytoid variant of eccrine sweat gland carcinoma of eyelids.

## Conclusions

Early diagnosis is paramount in histiocytoid eccrine sweat gland carcinoma of the eyelid. Radical surgery is the first choice treatment. A long follow-up is recommended, and bilateral involvement is possible.

## Conflicts of interest

The authors declare no conflicts of interest.
